# Interspecific Associations of Dominant Tree Species at Different Structural Levels and Community Stability in the Habitat of Endangered Plant *Hopea hainanensis* Merr. & Chun

**DOI:** 10.3390/plants14162546

**Published:** 2025-08-15

**Authors:** Shaocui He, Donghai Li, Xiaobo Yang, Dongling Qi, Naiyan Shang, Caiqun Liang, Rentong Liu, Chunyan Du, Hao Ding, Binglin Ye

**Affiliations:** 1 School of Ecology, Hainan University, Haikou 570228, China; 2 Rubber Research Institute, Sanya Research Institute, China Academy of Tropical Agricultural Sciences, Haikou 571101, China

**Keywords:** National Park of Hainan Tropical Rainforest, endangered species, forest layer structure, interspecific relationships, community stability

## Abstract

The endangered plant *Hopea hainanensis* serves as both an indicator and keystone species in tropical rainforests, and its survival status is influenced by the interspecific relationships among coexisting tree species within the community. To explore these relationships, species resource utilization patterns, and community succession dynamics within the endangered plant community, this study utilized survey data from the *Hopea hainanensis* community in the Bawangling and Jianfengling branches of the National Park of Hainan Tropical Rainforest. Various analytical methods were employed, including the Variance Ratio (*VR*) method, test statistic (*W*), *χ*^2^ test, Spearman’s rank correlation, and M. Godron’s stability analysis, to examine the interspecific associations among dominant tree species at different structural levels in the two regions and their effects on community stability. The results indicate that: (1) *Hopea hainanensis* is the dominant species in the medium tree layer in both study areas, while it functions as an associated species in other structural layers. (2) In communities where *Hopea hainanensis* is present in both Bawangling and Jianfengling, the dominant tree species across various structural layers generally show a non-significant positive association. (3) The results of the *χ*^2^ test and Spearman’s rank correlation test reveal that the interspecific associations across different structural layers of the *Hopea hainanensis* communities in both regions are predominantly non-significant. This suggests weak interspecific relationships and a high degree of species independence. The communities at different structural levels in both Bawangling and Jianfengling are in an unstable state, with ongoing dynamic adjustments to their internal tree species composition and structure. In terms of stability, the community stability across structural levels in these two regions follows the order: middle shrub layer > middle arbor layer > small arbor layer > large shrub layer. This study reveals the interspecific relationships, community succession status, and stability of dominant tree species at different structural levels in slope barrier communities across regions. These findings provide a theoretical basis for developing scientifically sound and reasonable protection strategies for slope barrier populations, as well as for the restoration and sustainable development of tropical rainforest vegetation.

## 1. Introduction

Interspecific relationships describe the interactions between different populations within a community, reflecting the connections and influences of plant populations. These relationships are key factors in determining the composition, structure, function, and dynamic changes of communities [1,2,3,4]. Analytical methods for studying interspecific relationships primarily involve two aspects: interspecific associations and interspecific correlations. Both concepts are used to describe the spatial distribution relationships among species within a community, but they differ in their research approaches [5]. Interspecific association primarily uses binary data on species presence or absence to qualitatively analyze whether species are associated [6], whereas interspecific correlation relies on quantitative indicators, such as abundance, coverage, and importance value, to numerically reflect the relationships among species [7]. Community stability is a critical characteristic for measuring the structure and function of a community, as it determines whether the community can persist and continue to develop. This stability largely depends on the interrelationships between species [8]. Investigating interspecific associations and community stability is crucial for understanding community structure, identifying trends in community succession, accurately determining the functions of various populations within the community, and their interactions with environmental factors and other species [9]. Furthermore, this research provides theoretical support for forest management, natural vegetation restoration, and biodiversity conservation. In recent years, extensive research has been conducted on interspecific interactions and community stability in plant communities across islands, deserts, and karst regions [10,11,12]. However, studies examining interspecific connectivity and stability at different structural levels within tropical rainforests—an ecosystem known for its extraordinarily high biodiversity and complex structure—remain relatively limited, particularly those focusing on endangered plant species. This research distinguishes itself from previous studies on tropical plant communities by specifically investigating the interactions between endangered species and other species [13]. Its innovation lies in uncovering the critical roles of these endangered species as connectivity nodes at various levels within the community, and in developing a relationship model that links “structural level—connectivity—stability.” These findings offer valuable insights into the coexistence mechanisms of endangered species in tropical rainforests and provide a reference for conservation strategies targeting endangered plants in similar ecosystems.

Hainan’s tropical rainforest is one of China’s most biodiverse regions, boasting the richest variety of tropical flora and the most comprehensive spectrum of tropical vegetation types. It is also a global hotspot for biodiversity conservation [14]. However, the expansion of *Hevea brasiliensis* (Willd. ex A. Juss.) Müll. Arg. and *Areca catechu* L. nut cultivation has exacerbated land reclamation, irrational land use, and deforestation, increasing the pressure on endangered plant species. *Hopea hainanensis*, a key species of the Dipterocarpaceae family and *Hopea* genus, is an indicator species of tropical rainforests. It is endemic to Hainan, China, and northern Vietnam [15,16]. In the 1990s, overexploitation of *Hopea hainanensis* due to its high value led to a sharp decline in its population. It has shifted from being a dominant species in these rainforests to a rare one [17]. Furthermore, severe limitations in recruitment from seedlings to saplings have resulted in a low number of mature individuals, making the conservation of existing *Hopea hainanensis* plants urgent [18]. While current research on *Hopea hainanensis* largely focuses on resource surveys, community structure, biological characteristics, and conservation genetics [19,20,21,22], studies examining the interspecific relationships of species at different structural levels within *Hopea hainanensis* communities have yet to be reported. Therefore, this study focuses on the endangered species *Hopea hainanensis* and its community in the Hainan tropical rainforest, systematically analyzing for the first time the interspecies association network and stability mechanisms across vertical stratification within this community.

The Bawangling and Jianfengling branches of the National Park of Hainan Tropical Rainforest represent the primary distribution areas for the wild population of *Hopea hainanensis*. Historically, primary forests have gradually been replaced by secondary forests, agricultural land, and plantations due to overexploitation, irrational land use, and deforestation, resulting in habitat loss and fragmentation. The micro-environmental changes caused by the destruction of the rainforest canopy further endanger the survival of *Hopea hainanensis*. As a keystone and indicator species in tropical rainforests, the long-term survival of *Hopea hainanensis* depends not only on its own adaptability but also on the overall stability of the community it inhabits. The vertical structure, density, and canopy closure of tropical rainforests far surpass those of temperate ones. Therefore, their restoration must involve rebuilding a multi-layered structure and ensuring high connectivity.

This study focuses on the *Hopea hainanensis* populations in the Bawangling and Jianfengling branches of Hainan Tropical Rainforest. Based on field investigations, we systematically analyzed the interspecific associations and community stability of dominant tree species at different structural levels within the communities where *Hopea hainanensis* is found. Field investigations were conducted employing methods such as the Variance Ratio (*VR*), *χ*^2^ test, Spearman rank correlation, and M. Godron stability analysis to address the following scientific questions: (1) What is the ecological status of *Hopea hainanensis* at different structural levels within its communities? (2) What are the patterns of interspecific associations among species at various structural levels in communities containing *Hopea hainanensis*? (3) How stable are the different structural levels of these communities? (4) Are there differences in the structure and function of *Hopea hainanensis* communities across different geographical regions (Bawangling and Jianfengling)?

## 2. Materials and Methods

### 2.1. Study Area

The Bawangling Branch (18°57′–19°11′ N, 109°03′–109°17′ E) and Jianfengling Branch (18°34′–18°52′ N, 108°44′–109°4′ E) of the National Park of Hainan Tropical Rainforest are located in the southwest of Hainan Island and are both influenced by a tropical monsoon climate. The elevation in Bawangling ranges from 50 to 1654 m, while in Jianfengling, it spans from 112 to 1654 m. These regions are rich in plant biodiversity and host nationally protected species, such as *Hopea hainanensis*, *Vatica mangachapoi*, and *Cephalotaxus hainanensis*. The average annual temperature in Bawangling is 23.6 °C, with an average annual precipitation of 1657 mm. The soils in this region are predominantly lateritic, resulting from the weathering of granite and limestone, and remain moist year-round. In Jianfengling, the average annual temperature is slightly higher, at 24.5 °C, with an average annual precipitation of 2265.8 mm. The soil types in the area include lateritic red, lateritic yellow, yellow, and dry red soils, with lateritic yellow and red soils being the most widespread [23,24].

### 2.2. Plot Setup and Survey Methodology

**Figure 1:**
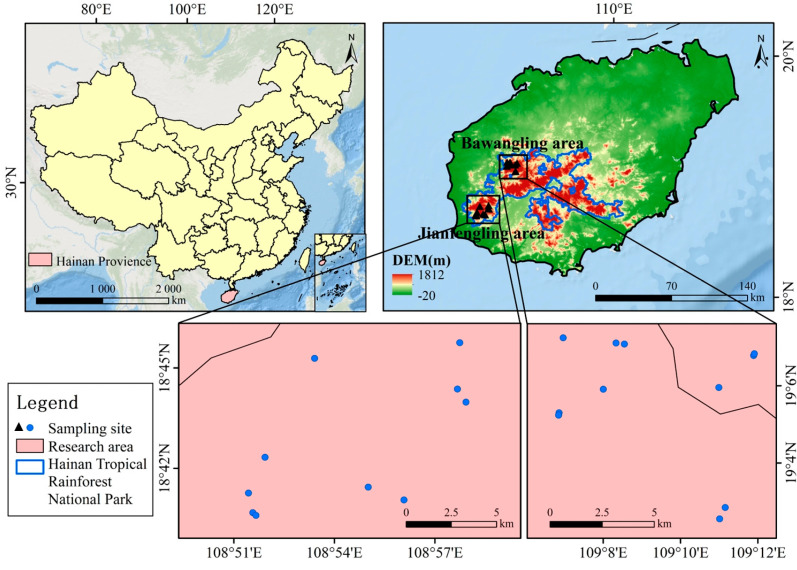
The distribution of *Hopea hainanensis* in the two regions, including the Bawangling and Jianfengling branches.

A study on *Hopea hainanensis* within the Bawangling and Jianfengling divisions was conducted from October to December 2024, based on multiple field surveys. Random sampling and typical plot methods were employed, with 20 × 20 m plots established around well-developed *Hopea hainanensis* trees (11 in Bawangling and 10 in Jianfengling), as shown in Figure 1. Each plot was divided into four 10 × 10 m tree quadrats, with additional subdivisions of five 5 × 5 m shrub quadrats and five 1 × 1 m herbaceous quadrats placed at the center and four corners. All trees, shrubs, and herbs within the quadrats were surveyed, and their species names, diameter at breast height (DBH), tree height, and crown widths were measured and recorded. Additionally, the altitude, latitude, longitude, canopy density, and slope of the plots were determined and documented.

### 2.3. Identification of Species

This study identified the species within the community where *Hopea hainanensis* is located, using “Flora of China”, “Flora Reipublicae Popularis Sinicae”, and “Pictorial Flora of Hainan” as reference materials [25,26].

### 2.4. Data Processing

#### 2.4.1. Stratification of Forest Layers and Calculation of Importance Values

The data from field surveys were categorized according to the methods of Wang GH et al. [27], with classifications into the following layers: middle arbor layer (25 m ≤ H < 8 m), small arbor layer (8 m ≤ H < 5 m), large shrub layer (5 m ≤ H < 2 m), middle shrub layer (2 m ≤ H < 0.5 m), and herbaceous layer (H < 0.5 m). No tree species from the large tree layer (H ≥ 25 m) were recorded in the community.

The importance values were calculated following the method proposed by Zhao Y et al. [28]. This study focused on the top 20 dominant tree species based on importance values from both the medium and small arbor layers, as well as the large and middle shrub layers, within the community where *Hopea hainanensis* is found. Due to the limited number of herbaceous species recorded, the herbaceous layer was not analyzed further.

#### 2.4.2. Overall Connectivity Test

The overall connectivity of the community was calculated following the method of Schluter et al. [29], using the variance ratio (*VR*) and test statistic (*W*) for analysis.

#### 2.4.3. Interspecific Connectivity and Interspecies Correlation Analysis

The interspecific associations of dominant tree species at various structural levels within the community were qualitatively analyzed using the *χ*^2^ test. A binary matrix (with 0 denoting the absence of a species in a quadrat and 1 indicating its presence) was constructed based on species’ occurrence within the quadrats. A 2 × 2 contingency table was then generated from this matrix [30]. Given the study’s use of discontinuous sampling, the *χ*^2^ statistic was calculated using Yates’ continuity correction, following the method proposed by Zhang JT et al. for analyzing interspecific associations [31].

The *χ*^2^ test can only provide a qualitative assessment of whether interspecific associations are significant, but it cannot definitively conclude that there is no association between species pairs when the *χ*^2^ test results are not significant [32]. Furthermore, the *χ*^2^ test does not distinguish differences in the strength of associations between different species pairs, nor does it clearly quantify the specific nature of interspecific associations. In contrast, this study utilizes the Spearman rank correlation test, which incorporates quantitative species indicators (importance values), to more accurately and objectively analyze the correlations among dominant tree species at different structural levels of the community. This approach effectively supplements and enhances the findings of the *χ*^2^ test.

#### 2.4.4. Community Stability Analysis

The stability of the community was analyzed using the improved M. Godron stability measurement method by Zheng YR [33].

### 2.5. Data Analysis

Data organization and calculation of key values were performed using Excel 2021, the *χ*^2^ test was conducted using the spaa package in R 4.4.2, and Spearman’s rank correlation test and the creation of a half-matrix plot were accomplished using Origin 2024.

## 3. Results

### 3.1. The Importance Value of Dominant Tree Species in Different Structural Layers of the Community Where Hopea hainanensis Is Located

As shown in Table 1, within the community where *Hopea hainanensis* is found in Bawangling, the tree layer holds the highest importance value for *Hopea hainanensis* (12.44), the small arbor layer for *Diospyros cathayensis* Steward (6.18), the large shrub layer for *Psychotria asiatica* L. (10.46), and the middle shrub layer for *Carpinus turczaninovii* Hance (12.43). In the community where *Hopea hainanensis* is located in Jianfengling, the tree layer again holds the highest importance value for *Hopea hainanensis* (5.75), the small arbor layer for *Croton laevigatus* Vahl (33.21), the large shrub layer for *Lasianthus lancifolius* Hook. f. (23.98), and the middle shrub layer for *Gymnosphaera podophylla* (Hook.) Copel. (25.47) (see Table 2). In both regions, the tree layer exhibits the highest importance value for *Hopea hainanensis*, signifying its dominant role as a constructive species in this layer, while it serves as an associated species in the other layers.

### 3.2. The Overall Association of Dominant Tree Species Across Different Structural Levels in the Community Where Hopea hainanensis Is Located

The variance ratio (*VR*) method was utilized to assess the overall connectivity among dominant species across different structural levels of the community. The significance of the results was tested using the *W-statistic*. As indicated in Table 3, in Bawangling, the *VR* values for the middle arbor layer, small arbor layer, large shrub layer, and middle shrub layer were all greater than 1. The *W* values fell within the range 20.95 (11) < *W* < 20.05 (11), suggesting a non-significant positive association in the overall connectivity of dominant tree species across various structural levels. Similarly, as presented in Table 4 for Jianfengling, the *VR* values for the middle arbor layer, small arbor layer, large shrub layer, and middle shrub layer were also greater than 1, with the *W* values falling within the range 20.95 (10) < *W* < 20.05 (10). This also indicates a non-significant positive association.

### 3.3. Interspecific Associations of Dominant Tree Species Across Different Structural Levels in the Hopea hainanensis Community

#### 3.3.1. Interspecific Connectivity

**Figure 2:**
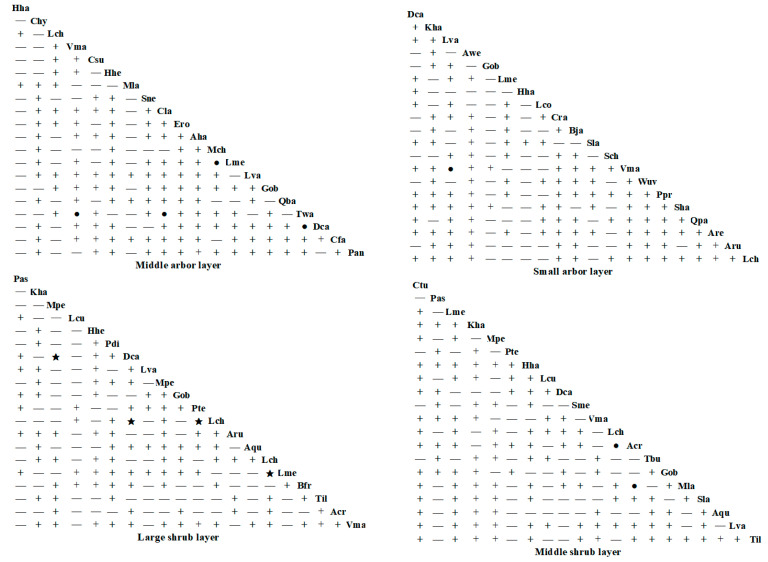
*χ*^2^ test of *Hopea hainanensis* species in different forest layers of the community in Bawangling. Note: + indicates a non-significant positive association; ● indicates a significant positive association; — indicates a non-significant negative association; ★ indicates a significant negative association. Hha: *Hopea hainanensis*; Chy: *Castanopsis hystrix*; Lch: *Litchi chinensis*; Vma: *Vatica mangachapoi*; Csu: *Canarium subulatum*; Hhe: *Heptapleurum heptaphyllum*; Mla: *Monoon laui*; Sne: *Syzygium nervosum*; Cla: *Croton laevigatus*; Ero: *Engelhardia roxburghiana*; Aha: *Adinandra hainanensis*; Mch: *Machilus chinensis*; Lme: *Lithocarpus megalophyllus*; Lva: *Litsea variabilis*; Gob: *Garcinia oblongifolia*; Tsi: *Toona sinensis*; Twa: *Tarennoidea wallichii*; Dca: *Diospyros cathayensis*; Cfa: *Castanopsis faberi*; Pan: *Pouteria annamensis*; Kha: *Koilodepas hainanense*; Awe: *Arenga westerhoutii*; Loc: *Lithocarpus corneus*; Cra: *Chionanthus ramiflorus*; Bja: *Bischofia javanica*; Sla: *Sterculia lanceolata*; Sch: *Syzygium chunianum*; Wuv: *Wendlandia uvariifolia*; Ppr: *Photinia prunifolia*; Sha: *Syzygium hancei*; Qpa: *Quercus patelliformis*; Are: *Actinodaphne reticulata*; Aru: *Alchornea rugosa*; Pas: *Psychotria asiatica*; Mpe: *Mallotus peltatus*; Lcu: *Lasianthus curtisii*; Pdi: *Psydrax dicocca*; Pte: *Prismatomeris tetrandra*; Lch: *Lasianthus chinensis*; Aqu: *Ardisia quinquegona*; Bfr: *Breynia fruticosa*; Til: *Taxotrophis ilicifolia*; Acr: *Ardisia crispa*; Ctu: *Carpinus turczaninovii*; sme: Saprosma merrillii; Tbu:Tabernaemontana bufalina.

The interspecific association test results for the dominant tree species in the middle arbor layer, small arbor layer, large shrub layer, and middle shrub layer of the community where *Hopea hainanensis* is located in Bawangling (see Figure 2) reveal the following: Out of 190 species pairs, the number of positively associated species pairs in the tree, small arbor, large shrub, and middle shrub layers are 126, 121, 97, and 118, respectively. These account for 66.32%, 63.68%, 51.05%, and 62.11% of the total species pairs in each layer. Conversely, the number of negatively associated species pairs in these layers is 64, 69, 93, and 72, accounting for 33.68%, 36.32%, 48.95%, and 37.89% of the total species pairs, respectively. In the middle arbor layer, there are four significantly positive associations: *Tarennoidea wallichii* (Hook. f.) Tirveng. & Sastre—*Vatica mangachapoi* Blanco, *Tarennoidea wallichii*—*Diospyros eriantha*, *Tarennoidea wallichii*—*Croton oblongifolius*, and *Machilus chinensis* (Champ. ex Benth.) Hemsl.—*Lithocarpus megalophyllus* Rehder & E. H. Wilson. In the large shrub layer, there is one significant positive association: *Litsea variabilis* Hemsl.*—Vatica mangachapoi*. In the large shrub layer, there are four significant negative associations: *Diospyros eriantha*—*Koilodepas hainanense* (Merr.) Croizat, *Diospyros eriantha*—*Lasianthus chinensis* (Champ. ex Benth.) Benth., *Prismatomeris tetrandra* (Roxb.) K. Schum.—*Lasianthus chinensis*, and *Lasianthus chinensis*—*Lithocarpus megalophyllus*. In the middle shrub layer, there are two significant positive species pairs: *Ardisia crispa* (Thunb.) A. DC.—*Litchi chinensis* Sonn. and *Ardisia crispa*—*Monoon laui.* (Merr.) B. Xue & R. M. K. Saunders The ratios of positive to negative associations for dominant species pairs in different structural layers of the *Hopea hainanensis* community in Bawangling are 1.97, 1.75, 1.04, and 1.64, respectively.

**Figure 3:**
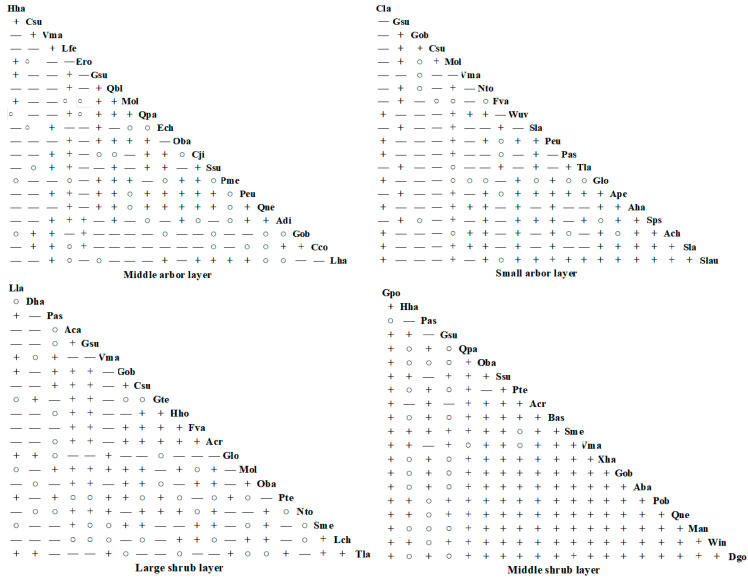
*χ*^2^ tests of *Hopea hainanensis* species in different forest layers of the community in Jianfengling. Note: + indicates a non-significant positive association; — indicates a non-significant negative association; and ○ indicates no association. Csu: *Canarium subulatum*; Lfe: *Lithocarpus fenzelianus*; Ero: *Engelhardia roxburghiana*; Gsu: *Gironniera subaequalis*; Qbl: *Quercus blakei*; Mol: *Maclurodendron oligophlebium*; Ech: *Endospermum chinense*; Oba: *Ormosia balansae*; Cji: *Castanopsis jianfenglingensis*; Ssu: *Schima superba*; Pme: *Pertusadina metcalfii*; Peu: *Pentaphylax euryoides*; Qne: *Quercus neglecta*; Adi: *Apodytes dimidiata*; Cco: *Cratoxylum cochinchinense*; Lha: *Lithocarpus handelianus*; Nto: *Nephelium topengii*; Fva: *Ficus vasculosa*; Wuv: *Wendlandia uvariifolia* Sla: *Symplocos lancifolia*; Tla: *Tarenna lancilimba*; Glo: *Gonocaryum lobbianum*; Ape: *Acronychia pedunculata*; Aha: *Adinandra hainanensis*; Sps: *Symplocos pseudobarberina*; Ach: *Amesiodendron chinense*; Slau: *Sarcosperma laurinum*; Lla: *Lasianthus lancifolius*; Dha: *Dehaasia hainanensis*; Aca: *Aidia canthioides*; Gte: *Gomphandra tetrandra*; Hho: *Hancea hookeriana*; Lch: *Lirianthe championii*; Tla: *Tarenna lancilimba*; Gpo: *Gymnosphaera podophylla*; Bas: *Buddleja asiatica*; Xha: *Xanthophyllum hainanense*; Aba: *Allomorphia balansae*; Pob: *Polyalthia obliqua*; Man: *Meliosma angustifolia*; Win: *Wikstroemia indica*; Dgo: *Dysoxylum gotadhora*.

For the community at Jianfengling (see Figure 3), the interspecific association test results for the dominant tree species in the middle arbor layer, small arbor layer, large shrub layer, and middle shrub layer show that, out of 190 species pairs, the number of positively associated pairs are 76, 90, 82, and 156, accounting for 40%, 47.37%, 43.16%, and 82.11% of the total species pairs, respectively. The number of negatively associated pairs is 78, 78, 68, and 7, accounting for 41.05%, 41.05%, 35.79%, and 3.68% of the total species pairs, respectively. The number of non-associated pairs is 36, 22, 40, and 27, accounting for 18.95%, 11.58%, 21.05%, and 14.21% of the total species pairs, respectively. In the Jianfengling community, the associations between dominant tree species at different structural levels are mostly non-significant, with the ratios of positive to negative associations being 0.97, 1.15, 1.21, and 22.29 in the middle arbor layer, small arbor layer, large shrub layer, and middle shrub layer, respectively.

Overall, the connectivity between dominant tree species at different structural levels within the communities of Bawangling and Jianfengling is largely characterized by non-significant associations, which aligns with the overall connectivity results. The *χ*^2^ test results indicate that most connections between dominant tree species across the different structural levels in both communities are non-significant, with only a few species pairs showing significant or highly significant connections.

#### 3.3.2. Interspecific Correlation

**Figure 4:**
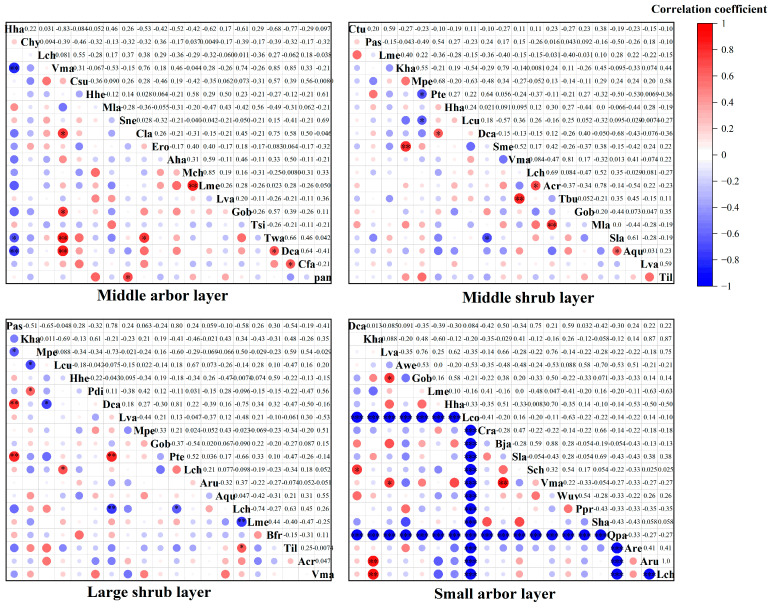
Spearman rank correlation test of *Hopea hainanensis* species in different forest layers of the community in Bawangling. Note: Hha: *Hopea hainanensis*; Chy: *Castanopsis hystrix*; Lch: *Litchi chinensis*; Vma: *Vatica mangachapoi*; Csu: *Canarium subulatum*; Hhe: *Heptapleurum heptaphyllum*; Mla: *Monoon laui*; Sne: *Syzygium nervosum*; Cla: *Croton laevigatus*; Ero: *Engelhardia roxburghiana*; Aha: *Adinandra hainanensis*; Mch: *Machilus chinensis*; Lme: *Lithocarpus megalophyllus*; Lva: *Litsea variabilis*; Gob: *Garcinia oblongifolia*; Tsi: *Toona sinensis*; Twa: *Tarennoidea wallichii*; Dca: *Diospyros cathayensis*; Cfa: *Castanopsis faberi*; Pan: *Pouteria annamensis*; Kha: *Koilodepas hainanense*; Awe: *Arenga westerhoutii*; Loc: *Lithocarpus corneus*; Cra: *Chionanthus ramiflorus*; Bja: *Bischofia javanica*; Sla: *Sterculia lanceolata*; Sch: *Syzygium chunianum*; Wuv: *Wendlandia uvariifolia*; Ppr: *Photinia prunifolia*; Sha: *Syzygium hancei*; Qpa: *Quercus patelliformis*; Are: *Actinodaphne reticulata*; Aru: *Alchornea rugosa*; Pas: *Psychotria asiatica*; Mpe: *Mallotus peltatus*; Lcu: *Lasianthus curtisii*; Pdi: *Psydrax dicocca*; Pte: *Prismatomeris tetrandra*; Lch: *Lasianthus chinensis*; Aqu: *Ardisia quinquegona*; Bfr: *Breynia fruticosa*; Til: *Taxotrophis ilicifolia*; Acr: *Ardisia crispa*; Ctu: *Carpinus turczaninovii*; Sme: Saprosma merrillii; Tbu:Tabernaemontana bufalina. *p* < 0.05 * *p* < 0.01 ** *p* < 0.001 ***.

As shown in Figure 4, the results of the Spearman rank correlation test reveal that within the community containing *Hopea hainanensis* in Bawangling, there are 190 species pairs composed of dominant tree species across various layers: middle arbor layer, small arbor layer, large shrub layer, and middle shrub layer. Among these, the number of positively correlated species pairs is 82, 60, 84, and 85, representing 43.16%, 31.58%, 44.21%, and 44.74% of the total species pairs, respectively. Conversely, the number of negatively correlated species pairs is 108, 128, 106, and 103, accounting for 56.84%, 63.37%, 55.79%, and 54.21% of the total species pairs, respectively. Additionally, there are 0, 2, 6, and 2 uncorrelated species pairs, accounting for 0%, 1.05%, 3.16%, and 1.05% of the total, respectively. In terms of significant correlations, the middle arbor layer, small arbor layer, large shrub layer, and middle shrub layer show 9, 6, 6, and 6 species pairs with significant positive correlations at the highly significant (*p* < 0.01) or significant (*p* < 0.05) levels, while the number of species pairs with negative correlations are 3, 38, 6, and 3, respectively. Notably, *Hopea hainanensis* exhibits significant negative correlations with three tree species: *Vatica mangachapoi*, *Diospyros cathayensis*, and *Tarennoidea wallichii*, as well as extremely significant negative correlations with *Lithocarpus corneus* (Lour.) Rehder and *Cyclobalanopsis patelliformis* (Chun) Y. C. Hsu & H. W. Jen.

**Figure 5:**
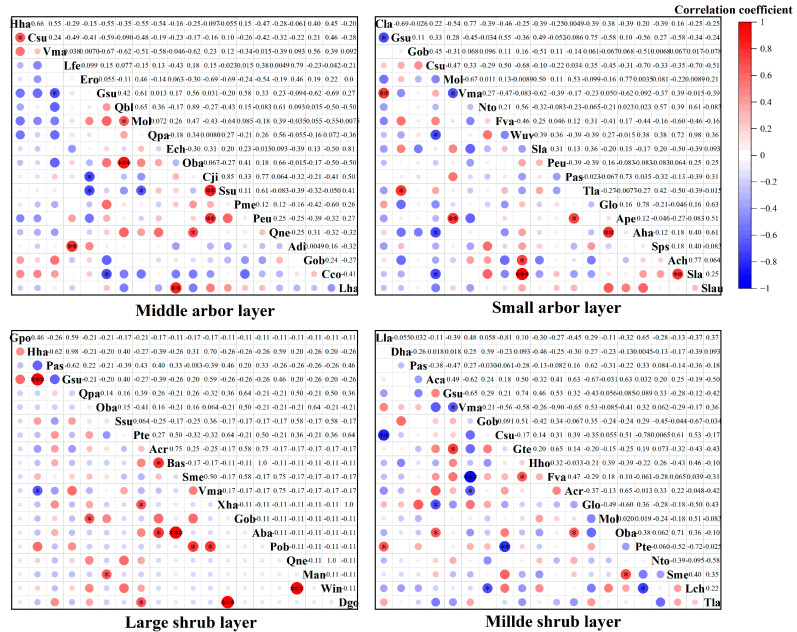
Spearman rank correlation test of *Hopea hainanensis* species in different forest layers of the community in Jianfengling. Note: Csu: *Canarium subulatum*; Lfe: *Lithocarpus fenzelianus*; Ero: *Engelhardia roxburghiana*; Gsu: *Gironniera subaequalis*; Qbl: *Quercus blakei*; Mol: *Maclurodendron oligophlebium*; Ech: *Endospermum chinense*; Oba: *Ormosia balansae*; Cji: *Castanopsis jianfenglingensis*; Ssu: *Schima superba*; Pme: *Pertusadina metcalfii*; Peu: *Pentaphylax euryoides*; Qne: *Quercus neglecta*; Adi: *Apodytes dimidiata*; Cco: *Cratoxylum cochinchinense*; Lha: *Lithocarpus handelianus*; Nto: *Nephelium topengii*; Fva: *Ficus vasculosa*; Wuv: *Wendlandia uvariifolia* Sla: *Symplocos lancifolia*; Tla: *Tarenna lancilimba*; Glo: *Gonocaryum lobbianum*; Ape: *Acronychia pedunculata*; Aha: *Adinandra hainanensis*; Sps: *Symplocos pseudobarberina*; Ach: *Amesiodendron chinense*; Slau: *Sarcosperma laurinum*; Lla: *Lasianthus lancifolius*; Dha: *Dehaasia hainanensis*; Aca: *Aidia canthioides*; Gte: *Gomphandra tetrandra*; Hho: *Hancea hookeriana*; Lch: *Lirianthe championii*; Tla: *Tarenna lancilimba*; Gpo: *Gymnosphaera podophylla*; Bas: *Buddleja asiatica*; Xha: *Xanthophyllum hainanense*; Aba: *Allomorphia balansae*; Pob: *Polyalthia obliqua*; Man: *Meliosma angustifolia*; Win: *Wikstroemia indica*; Dgo: *Dysoxylum gotadhora. p* < 0.05 * *p* < 0.01 ** *p* < 0.001 ***.

As shown in Figure 5, within the community of *Hopea hainanensis* exalata in Jianfengling, among the same 190 species pairs across the middle arbor layer, small arbor layer, large shrub layer, and middle shrub layer, the number of positively correlated species pairs is 88, 89, 82, and 61, accounting for 46.32%, 46.84%, 43.16%, and 32.11% of the total species pairs, respectively. The number of negatively correlated species pairs is 101, 101, 108, and 129, which account for 53.16%, 53.16%, 56.84%, and 67.89%, respectively. Within these layers, the number of species pairs showing significant positive correlations at the extremely significant (*p* < 0.001) or significant (*p* < 0.05) levels is 5, 8, 5, and 12, respectively, while the negatively correlated pairs are 5, 5, 8, and 1, respectively. One noteworthy finding is that *Hopea hainanensis* and Gironniera show a significant positive correlation.

In summary, the interspecific correlations between most pairs of dominant tree species across different structural layers in communities where *Hopea hainanensis* is distributed in both regions appear to be weak, as indicated by the *χ*^2^ test, which aligns with the Spearman correlation results.

### 3.4. The Community Stability of Different Structural Levels in the Community Where Hopea hainanensis Is Located

**Figure 6:**
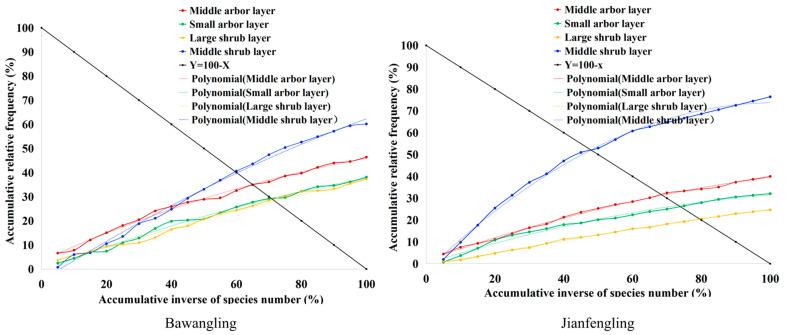
The stability and characterization of each forest layer in the communities where *Hopea hainanensis* of the two regions is located.

As shown in Figure 6, the Godron stability index curves for the dominant tree species in the tree layer, small tree layer, large shrub layer, and medium shrub layer of the community where Hopea hainanensis is found in Bawangling are as follows: middle arbor layer: Y = −0.0018X^2^ + 0.6001X + 3.597 (R^2^ = 0.9955), small arbor layer: Y = −0.0013X^2^ + 0.5177X − 0.7026 (R^2^ = 0.9949), large shrub layer: Y = −0.0005X^2^ + 0.4119X + 0.9377 (R^2^ = 0.9932), and middle shrub layer: Y = −0.0018X^2^ + 0.8471X − 4.7448 (R^2^ = 0.996). The intersection points of these curves with the linear equation Y = 100 − X are (65, 35), (70.27, 29.73), (71.9, 28.1), and (60.25, 39.75). The Euclidean distances from the stability point (20, 80) are 63.64, 71.09, 73.40, and 56.92, respectively. Based on these findings, it can be concluded that the stability of the community at different structural levels follows this order: middle shrub layer > middle arbor layer > small arbor layer > large shrub layer.

In the community located on Jianfeng Ridge, where *Hopea hainanensis* is found, the Godron stability index curves for the dominant tree species in different layers of the vegetation are as follows: middle arbor layer (Y = −0.0017X^2^ + 0.5549X + 1.5173, R^2^ = 0.9981), small arbor layer (Y = −0.0018X^2^ + 0.4987X + 0.0403, R^2^ = 0.9893), large shrub layer (Y = −0.0005X^2^ + 0.3138X − 1.0824, R^2^ = 0.9986), and middle shrub layer (Y = −0.0074X^2^ + 1.5059X − 3.1527, R^2^ = 0.996). The intersection points of these curves with the linear equation Y = 100 − X are (68.47, 31.53), (73.11, 26.89), (79.3, 20.7), and (47.91, 52.09). The Euclidean distances from the stable point (20, 80) are 68.55, 75.11, 83.86, and 39.47. Based on these results, it can be concluded that the stability of the community at different structural levels follows the order: middle shrub layer > middle arbor layer > small arbor layer > large shrub layer.

## 4. Discussion

### 4.1. The Importance Value of Dominant Tree Species at Different Structural Levels in the Community Where Hopea hainanensis Is Located

The importance value is commonly used to quantify the relative significance and ecological dominance of species within a community. A higher importance value indicates greater ecological dominance [34]. This study found that *Hopea hainanensis* occupies a dominant species position in the middle tree layer at both Bawangling and Jianfengling, suggesting its strong resource competition capabilities and ecological dominance within this layer. However, *Hopea hainanensis* is only an associated species in other structural layers (such as the small arbor layer and shrub layers). This vertical distribution pattern indicates that the population renewal of *Hopea hainanensis* may face bottlenecks, and its absence from the understory could hinder natural regeneration. Interspecific competition pressures at different levels may vary significantly. For example, high importance value species in the shrub layer, such as *Psychotria asiatica and Lasianthus lancifolius* Hook. f., could exert strong competition on the seedling regeneration of *Hopea hainanensis*. Moreover, the significant difference in the importance value of *Hopea hainanensis* between the two study areas (with Bawangling showing a significantly higher value than Jianfengling) may reflect differences in the adaptability of this species under varying habitat conditions.

### 4.2. The Overall Connectivity of Dominant Tree Species Across Different Structural Levels in the Community Where Hopea hainanensis Is Located

First, the non-significant positive association suggests that there may be weak niche overlap or similar environmental requirements (such as light, moisture, or soil nutrients) among the dominant species in the community. This could lead to some degree of co-occurrence in their spatial distribution [35]. However, the lack of statistical significance indicates that community assembly is likely influenced more by environmental heterogeneity or stochastic processes than by strong interspecific interactions (mutualism or competitive exclusion) [36]. This aligns with the high species diversity typical of tropical forest communities, where resource allocation tends to be relatively balanced, thereby reducing the competitive advantage of any single species. Second, from a community succession perspective, non-significant associations are often observed in the early or middle stages of succession. This aligns with findings from studies on the endangered plant species *Abies beshanzuensis* M. H. Wu [34], suggesting that *Hopea hainanensis* communities may still be in a structurally unstable phase and are vulnerable to external disturbances. It is noteworthy that *Hopea hainanensis* is predominantly distributed in the midstory tree layer in both Bawangling and Jianfengling, while its associated species primarily occupy the understory tree layer and shrub layer. Field investigations have shown that *Hopea hainanensis* prefers to grow in heterogeneous habitats, such as streamsides, gullies, and upper slopes with high rock exposure. These microenvironments often result in discontinuous canopy coverage in the midstory tree layer, which in turn affects the light and moisture conditions of the lower layers (e.g., the shrub layer). This creates gradient differences in the stability of various structural layers (middle shrub layer > middle arbor layer > small tree layer > large-shrub layer). This pattern contrasts with the continuous vertical structure of typical tropical primary forests, further supporting the long-term and complex nature of secondary-forest restoration.

### 4.3. Interspecific Associations of Dominant Tree Species Across Different Structural Layers in the Community Where Hopea hainanensis Is Present

Interspecific association refers to the spatial correlation between different species, reflecting the mutual influence of species and their community dynamics [37,38]. Studies have shown that as a community undergoes succession, its structure and composition become gradually stabilized. The ratio of positive to negative associations among species, which is relatively low in the early stages of succession, tends to shift toward a predominantly positive association in later stages. This shift results in a larger ratio of positive to negative associations, indicating progression toward a climax community [39]. In this study, Spearman’s rank correlation test revealed that in the different structural layers of communities where *Hopea hainanensis* is found in Bawangling and Jianfengling, the number of negative associations among dominant tree species is generally higher than the number of positive associations. However, the overall connectivity across these structural layers showed an insignificant positive correlation. This result aligns with Liu RH’s study on the interspecific associations of major woody plant species in the riparian zone of the Li River [30]. This suggests that niche differentiation has occurred among the major populations at different structural levels during the succession of the community where *Hopea hainanensis* is located. Mutualistic interactions have developed between some species, leading to the formation of relatively stable community structures. Additionally, the connectivity between dominant tree species at different structural levels within the communities where *Hopea hainanensis* is found in both regions is primarily characterized by non-significant connections. Only a few species pairs exhibited highly significant associations. This indicates that the connectivity between species at different structural levels is relatively weak, and the independence of each species is relatively high. This phenomenon suggests that the communities are in the early or middle stages of unstable succession, primarily due to environmental heterogeneity, resource competition, and disturbances from environmental factors. Furthermore, *Hopea hainanensis* is predominantly distributed in the middle tree and herbaceous layers, suggesting that the current individuals of *Hopea hainanensis* are primarily seedlings and large trees, with very few young or small trees. This conclusion aligns with the survey results, mainly due to severe recruitment limitations during the development of *Hopea hainanensis* seedlings and the influence of complex environmental factors [40].

The positive correlation between species pairs stems from their similar biological characteristics and close ecological adaptability to the environment, which reflects, to some extent, their similar resource utilization [41,42]. For example, there is a highly significant positive association between *Hopea hainanensis* and *Gironniera* in the small shrub layer of Jianfengling, indicating a strong synergistic relationship or niche complementarity between the two species. This suggests a high overlap in resource utilization, such as light, with minimal competition. Conversely, negative correlations between species pairs reflect exclusion phenomena, primarily due to differences in biological characteristics, varying environmental preferences, or direct competition. In the Bawangling area, the dominant tree layer consisting of *Hopea hainanensis—Vatica mangachapoi* and *Hopea hainanensis—Diospyros eriantha*, along with the sub-dominant tree layer formed by *Hopea hainanensis—Lithocarpus polystachyus* and *Hopea hainanensis—Cyclobalanopsis patelliformis*, exhibits highly significant negative associations. This indicates that these species have high resource demands and that there is intense competition or ecological niche exclusion among them. *Hopea hainanensis* directly competes with these species for resources.

### 4.4. The Stability of Different Structural Levels in the Community Where Hopea hainanensis Is Located

The analysis results based on the Godron stability index in this study indicate gradient differences in the stability of *Hopea hainanensis* communities across various structural levels in Bawangling and Jianfengling (middle shrub layer > middle arbor layer > small arbor layer > large shrub layer). This pattern reflects the ecological mechanisms of the vertical structure of tropical montane rainforests. Specifically, the middle shrub layer (Bawangling Euclidean distance 56.92, Jianfengling 39.47) demonstrates optimal stability, attributed to its strong shade tolerance and balanced resource allocation, while the large shrub layer (Bawangling Euclidean distance 73.40, Jianfengling 83.86), acting as an ecological transition zone, exhibits the highest vulnerability. This stable pattern suggests that the *Hopea hainanensis* communities in both regions are in the early to middle stages of succession and have not yet reached the climax community stage, which is consistent with the findings of Li KJ et al. regarding the *Ormosia microphylla* Merr. & H. Y. Chen community [43]. Both Bawangling and Jianfengling have been affected by deforestation due to agroforestry practices. However, Jianfengling has experienced more severe primary forest loss due to historically intensive agricultural development, such as rubber plantation development. Additionally, *Hopea hainanensis* in the Jianfengling area faces higher logging pressures, with existing populations largely scattered, while Bawangling retains relatively contiguous habitats. Although the stability of most structural levels in Bawangling is superior to that in Jianfengling, the stability of its shrub layer is slightly lower. This may reflect the influence of microhabitat differences on community construction processes in the two areas. The stability of the shrub layer, especially the middle shrub layer, is crucial for the growth of *Hopea hainanensis*. Its balanced resource allocation provides a shaded environment for the regeneration of *Hopea hainanensis* seedlings and buffers disturbances caused by canopy damage by maintaining favorable microclimatic conditions. This plays a key role in the restoration of Hainan’s tropical rainforest vegetation, particularly its unique endangered species.

## 5. Conclusions

In summary, (1) the overall connectivity among dominant tree species at different structural levels in the communities where Bawangling and Jianfengling *Hopea hainanensis* are located is a non-significant positive association, indicating that community succession is in a relatively unstable stage and susceptible to external factor disturbances; (2) the associations among dominant tree species at different structural levels in the communities where Bawangling and Jianfengling *Hopea hainanensis* are located are predominantly non-significant, with weak connectivity among species and strong independence of each species; and (3) the communities at different structural levels where Bawangling and Jianfengling *Hopea hainanensis* are located are in an unstable state, with the internal tree species composition and structure undergoing continuous adjustment and evolution. In conclusion, scientific and effective measures should be taken to protect the *Hopea hainanensis* population. In situ conservation can involve artificial tending and the rational use of species with similar ecological characteristics. During thinning, *Diospyros cathayensis* in the arbor layer and *Lithocarpus corneus* and *Cyclobalanopsis patelliformis* in the subarbor layer are fully utilized to optimize resource allocation within the community, allowing species to coexist. This provides a scientific basis for the conservation of *Hopea hainanensis* populations and the restoration of tropical rainforest vegetation.

## Figures and Tables

**Table 1:** Importance values of dominant tree species in different forest layers of the community with *Hopea hainanensis* in Bawangling.

Levels	Ranking	Latin Name	Abbreviation	Importance Value
Middle arbor layer	1	*Hopea hainanensis*	Hha	12.441
	2	*Castanopsis hystrix*	Chy	4.492
	3	*Litchi chinensis*	Lch	3.813
	4	*Vatica mangachapoi*	Vma	3.784
	5	*Canarium subulatum*	Csu	3.153
	6	*Heptapleurum heptaphyllum*	Hhe	2.792
	7	*Monoon laui*	Mla	2.728
	8	*Syzygium nervosum*	Sne	2.427
	9	*Croton laevigatus*	Cla	2.173
	10	*Engelhardia roxburghiana*	Ero	2.087
	11	*Adinandra hainanensis*	Aha	2.015
	12	*Machilus chinensis*	Mch	1.931
	13	*Lithocarpus megalophyllus*	Lme	1.872
	14	*Litsea variabilis*	Lva	1.841
	15	*Garcinia oblongifolia*	Gob	1.823
	16	*Toona sinensis*	Tsi	1.755
	17	*Tarennoidea wallichii*	Twa	1.744
	18	*Diospyros cathayensis*	Dca	1.737
	19	*Castanopsis faberi*	Cfa	1.716
	20	*Pouteria annamensis*	Pan	1.703
Small arbor layer	1	*Diospyros cathayensis*	Dca	6.175
	2	*Koilodepas hainanense*	Kha	4.026
	3	*Litsea variabilis*	Lva	3.788
	4	*Arenga westerhoutii*	Awe	3.747
	5	*Garcinia oblongifolia*	Gob	3.459
	6	*Lithocarpus megalophyllus*	Lme	3.040
	7	*Hopea hainanensis*	Hha	2.817
	8	*Lithocarpus corneus*	Lco	2.455
	9	*Chionanthus ramiflorus*	Cra	2.450
	10	*Bischofia javanica*	Bja	2.449
	11	*Sterculia lanceolata*	Sla	2.447
	12	*Syzygium chunianum*	Sch	2.235
	13	*Vatica mangachapoi*	Vma	1.835
	14	*Wendlandia uvariifolia*	Wuv	1.780
	15	*Photinia prunifolia*	Ppr	1.669
	16	*Syzygium hancei*	Sha	1.554
	17	*Quercus patelliformis*	Qpa	1.544
	18	*Actinodaphne reticulata*	Are	1.521
	19	*Alchornea rugosa*	Aru	1.501
	20	*Litchi chinensis*	Lch	1.409
Large shrub layer	1	*Psychotria asiatica*	Pas	10.457
	2	*Koilodepas hainanense*	Kha	7.704
	3	*Mallotus peltatus*	Mpe	4.340
	4	*Lasianthus curtisii*	Lcu	3.337
	5	*Heptapleurum heptaphyllum*	Hhe	3.028
	6	*Psydrax dicocca*	Pdi	2.803
	7	*Diospyros cathayensis*	Dca	2.700
	8	*Litsea variabilis*	Lva	2.533
	9	*Maesa perlarius*	Mpe	2.253
	10	*Garcinia oblongifolia*	Gob	2.058
	11	*Prismatomeris tetrandra*	Pte	1.819
	12	*Lasianthus chinensis*	Lch	1.796
	13	*Alchornea rugosa*	Aru	1.715
	14	*Ardisia quinquegona*	Aqu	1.646
	15	*Litchi chinensis*	Lch	1.640
	16	*Lithocarpus megalophyllus*	Lme	1.572
	17	*Breynia fruticosa*	Bfr	1.561
	18	*Taxotrophis ilicifolia*	Til	1.490
	19	*Ardisia crispa*	Acr	1.489
	20	*Vatica mangachapoi*	Vma	1.366
Middle shrub layer	1	*Carpinus turczaninovii*	Ctu	12.433
	2	*Psychotria asiatica*	Pas	10.637
	3	*Lithocarpus megalophyllus*	Lme	8.859
	4	*Koilodepas hainanense*	Kha	6.969
	5	*Mallotus peltatus*	Mpe	4.231
	6	*Prismatomeris tetrandra*	Pte	3.524
	7	*Hopea hainanensis*	Hha	3.513
	8	*Lasianthus curtisii*	Lcu	2.943
	9	*Diospyros cathayensis*	Dca	2.765
	10	*Saprosma merrillii*	Sme	2.721
	11	*Vatica mangachapoi*	Vma	2.536
	12	*Litchi chinensis*	Lch	2.037
	13	*Ardisia crispa*	Acr	1.971
	14	*Tabernaemontana bufalina*	Tbu	1.952
	15	*Garcinia oblongifolia*	Gob	1.725
	16	*Monoon laui*	Mla	1.479
	17	*Sterculia lanceolata*	Sla	1.357
	18	*Ardisia quinquegona*	Aqu	1.311
	19	*Litsea variabilis*	Lva	1.228
	20	*Taxotrophis ilicifolia*	Til	1.212

**Table 2:** Importance values of dominant tree species in different forest layers of the community with *Hopea hainanensis* in Jianfengling.

Levels	Ranking	Latin Name	Abbreviation	Importance Value
Middle arbor layer	1	*Hopea hainanensis*	Hha	5.749
	2	*Canarium subulatum*	Csu	4.210
	3	*Vatica mangachapoi*	Vma	4.113
	4	*Lithocarpus fenzelianus*	Lfe	3.584
	5	*Engelhardia roxburghiana*	Ero	3.157
	6	*Gironniera subaequalis*	Gsu	3.058
	7	*Quercus blakei*	Qbl	2.917
	8	*Maclurodendron oligophlebium*	Mol	2.860
	9	*Quercus patelliformis*	Qpa	2.719
	10	*Endospermum chinense*	Ech	2.615
	11	*Ormosia balansae*	Oba	2.548
	12	*Castanopsis jianfenglingensis*	Cji	2.499
	13	*Schima superba*	Ssu	2.464
	14	*Pertusadina metcalfii*	Pme	2.104
	15	*Pentaphylax euryoides*	Peu	1.846
	16	*Quercus neglecta*	Qne	1.677
	17	*Apodytes dimidiata*	Adi	1.630
	18	*Garcinia oblongifolia*	Gob	1.515
	19	*Cratoxylum cochinchinense*	Cco	1.425
	20	*Lithocarpus handelianus*	Lha	1.414
Small arbor layer	1	*Croton laevigatus*	Cla	33.211
	2	*Gironniera subaequalis*	Gsu	3.160
	3	*Garcinia oblongifolia*	Gob	2.649
	4	*Canarium subulatum*	Csu	2.599
	5	*Maclurodendron oligophlebium*	Mol	2.331
	6	*Vatica mangachapoi*	Vma	1.641
	7	*Nephelium topengii*	Nto	1.295
	8	*Ficus vasculosa*	Fva	1.272
	9	*Wendlandia uvariifolia*	Wuv	1.265
	10	*Symplocos lancifolia*	Sla	1.226
	11	*Pentaphylax euryoides*	Peu	1.178
	12	*Psychotria asiatica*	Pas	1.073
	13	*Tarenna lancilimba*	Tla	0.996
	14	*Gonocaryum lobbianum*	Glo	0.892
	15	*Acronychia pedunculata*	Ape	0.856
	16	*Adinandra hainanensis*	Aha	0.789
	17	*Symplocos pseudobarberina*	Sps	0.784
	18	*Amesiodendron chinense*	Ach	0.752
	19	*Sterculia lanceolata*	Sla	0.750
	20	*Sarcosperma laurinum*	Sla	0.675
Large shrub layer	1	*Lasianthus lancifolius*	Lla	23.975
	2	*Dehaasia hainanensis*	Dha	8.985
	3	*Psychotria asiatica*	Pas	3.230
	4	*Aidia canthioides*	Aca	2.842
	5	*Gironniera subaequalis*	Gsu	1.748
	6	*Vatica mangachapoi*	Vma	1.501
	7	*Garcinia oblongifolia*	Gob	1.495
	8	*Canarium subulatum*	Csu	1.352
	9	*Gomphandra tetrandra*	Gte	1.197
	10	*Hancea hookeriana*	Hho	1.118
	11	*Ficus vasculosa*	Fva	1.094
	12	*Ardisia crispa*	Acr	1.081
	13	*Gonocaryum lobbianum*	Glo	0.923
	14	*Maclurodendron oligophlebium*	Mol	0.877
	15	*Ormosia balansae*	Oba	0.864
	16	*Prismatomeris tetrandra*	Pte	0.821
	17	*Nephelium topengii*	Nto	0.819
	18	*Saprosma merrillii*	Sme	0.795
	19	*Lirianthe championii*	Lch	0.792
	20	*Tarenna lancilimba*	Tla	0.788
Middle shrub layer	1	*Gymnosphaera podophylla*	Gpo	25.469
	2	*Hopea hainanensis*	Hha	8.908
	3	*Psychotria asiatica*	Pas	6.757
	4	*Gironniera subaequalis*	Gsu	4.854
	5	*Quercus patelliformis*	Qpa	4.566
	6	*Ormosia balansae*	Oba	4.282
	7	*Schima superba*	Ssu	3.789
	8	*Prismatomeris tetrandra*	Pte	3.717
	9	*Ardisia crispa*	Acr	3.427
	10	*Buddleja asiatica*	Bas	2.965
	11	*Saprosma merrillii*	Sme	2.493
	12	*Vatica mangachapoi*	Vma	2.448
	13	*Xanthophyllum hainanense*	Xha	1.823
	14	*Garcinia oblongifolia*	Gob	1.775
	15	*Allomorphia balansae*	Aba	1.370
	16	*Polyalthia obliqua*	Pob	1.326
	17	*Quercus neglecta*	Qne	1.306
	18	*Meliosma angustifolia*	Man	1.306
	19	*Wikstroemia indica*	Win	1.306
	20	*Dysoxylum gotadhora*	Dgo	1.306

**Table 3:** Overall connectivity of dominant tree species in different forest strata of *Hopea hainanensis* in Bawangling.

Forest Stratum	Variance Ratio (*VR*)	Test Statistic (*W*)	*χ*^2^ Critical Value	Test Results
Middle arbor layer	1.73	19.03	(4.58, 19.68)	Non-significant positive association
Small arbor layer	1.40	11.23	(4.58, 19.68)	Non-significant positive association
Large shrub layer	1.11	12.21	(4.58, 19.68)	Non-significant positive association
Middle shrub layer	1.46	16.02	(4.58, 19.68)	Non-significant positive association

**Table 4:** Overall connectivity of dominant tree species in different forest strata of *Hopea hainanensis* in Jianfengling.

Forest Stratum	Variance Ratio (*VR*)	Test Statistic (*W*)	*χ*^2^ Critical Value	Test Results
Middle arbor layer	1.07	10.72	(3.94, 18.31)	Non-significant positive association
Small arbor layer	1.66	16.60	(3.94, 18.31)	Non-significant positive association
Large shrub layer	1.71	17.08	(3.94, 18.31)	Non-significant positive association
Middle shrub layer	1.62	16.23	(3.94, 18.31)	Non-significant positive association

## Data Availability

Data are contained within the article.
